# Draft Genome Sequence of Tenacibaculum haliotis Strain RA3-2^T^, Isolated from Korean Wild Abalone (Haliotis discus *hannai*)

**DOI:** 10.1128/mra.01025-22

**Published:** 2023-02-27

**Authors:** Ruben Avendaño-Herrera, Mónica Saldarriaga-Córdoba, Henry Araya-León, Rute Irgang

**Affiliations:** a Universidad Andrés Bello, Laboratorio de Patología de Organismos Acuáticos y Biotecnología Acuícola, Facultad de Ciencias de la Vida, Viña del Mar, Chile; b Centro FONDAP, Interdisciplinary Center for Aquaculture Research (INCAR), Universidad Andrés Bello, Viña del Mar, Chile; c Centro de Investigación Marina Quintay (CIMARQ), Universidad Andrés Bello, Valparaíso, Chile; d Centro de Investigación en Recursos Naturales y Sustentabilidad, Universidad Bernardo O'Higgins, Santiago, Chile; Montana State University

## Abstract

Here, we present the draft genome sequence of Tenacibaculum haliotis strain RA3-2^T^ (i.e., KCTC 52419^T^ and NBRC 112382^T^), isolated from Korean wild abalone (Haliotis discus *hannai*). As the only strain for this *Tenacibaculum* species worldwide, the information is of use for comparative genomic analyses delineating *Tenacibaculum* species.

## ANNOUNCEMENT

Most *Tenacibaculum* isolates are associated with fish (1). Two mollusk-derived species have been described, Tenacibaculum crassostreae from the Pacific oyster (Crassostrea gigas) (2) and Tenacibaculum haliotis from wild abalone (Haliotis discus *hannai*) (3).

Until now, no sequenced genome existed for the *T. haliotis* strain, RA3-2^T^. A sample of RA3-2^T^ was obtained from the Japanese Biological Resource Centre in glass ampoules (L-dried). The strain was grown on marine agar 2216 (BD Difco). The plates were incubated at 20°C for 3 days (3). Pure cultures were stored at −80°C in marine broth supplemented with 10% glycerol (vol/vol).

DNA was extracted from three colonies using the InstaGene matrix (Bio-Rad) per the manufacturer’s instructions. The 16S rRNA gene was PCR amplified using the universal primer pair 27F and 1492R (4). The amplicon was sequenced by Macrogen (Seoul, South Korea). The nearly complete 16S rRNA gene sequence comprised 1,391 nucleotides (see “Data availability,” below) and revealed 99.86% identity to that of *T. haliotis* RA3-2^T^ (GenBank accession number NR_158003).

The NucleoSpin soil kit (Macherey-Nagel GmbH & Co. KG), purified genomic DNA, and the NanoDrop Lite spectrophotometer were used to measure the DNA concentration (Thermo Fisher Inc.). The genome was sequenced by Macrogen. A DNA library was prepared using the TruSeq Nano DNA kit and sequenced using the Illumina NovaSeq 6000 platform (sequencing by synthesis; 2 × 150-bp, 300-cycle kit) to generate 31,942,898 paired-end reads with an average length of 151 bp.

The raw Illumina sequence data were quality checked (FastQC v.0.11.9; https://www.bioinformatics.babraham.ac.uk/projects/fastqc/). The next-generation sequencing reads were preprocessed using Geneious Prime v.2020.2.2 software (5): (i) reads were paired using the “set paired reads” function (insert size, 350 bp); (ii) poor-quality bases were trimmed using the BBDuk Trimmer plugin (removing reads with a size of <20 bp and quality score of <20), with paired-end read overlaps trimmed to ensure complete adapter removal; (iii) coverage was normalized by down-sampling the reads in high-depth genome areas using BBNorm with the “error correct” and “normalize reads” functions; and (iv) duplicate reads were removed using Dedupe. *De novo* genome assembly without a reference genome was carried out at the scaffold level using the Geneious assembler with default settings, and the quality was checked using QUAST v.5.2.0 (6). The assembly generated 29 scaffolds and 32 contigs (*N*_50_, 332,448 bp), with a total of 2,977,857 bp and 30.7% G+C content. The quantitated assembly quality indicated a minimum contig length of 1,346 bp, a maximum length of 486,103 bp, and an average length of 102,684 bp. The draft genome sequence contained 2,728 coding DNA sequences (CDSs) with protein, 2,794 genes, 8 pseudogenes, and 61 RNAs (48 tRNAs, 9 rRNAs, and 4 other RNAs), as determined by the NCBI Prokaryotic Genome Annotation Pipeline (PGAP) v.6.2 (https://github.com/ncbi/pgap) using the best-placed reference protein set, with GeneMarkS-2+ as the annotation method. The predicted tRNAs were verified using tRNAscan-SE v.2.0 (7).

Using *in silico* genome analysis, genes were discovered with predicted associations to secretion systems (type I [T1SS] and T9SS), as detected using TXSScan (MacSyFinder v.2; http://mobyle.pasteur.fr/cgi-bin/portal.py#forms::txsscan). Iron-related protein families were identified (heme iron transport, iron transport, siderophore synthesis, siderophore transport, transcriptional regulation, and iron storage) using FeGenie v.1.0 (8). Using the Resistance Gene Identifier v.6.0.0 incorporated with the Comprehensive Antibiotic Resistance Database v.3.2.5 (9), no antibiotic-resistance genes were found. Hemolysis, immune evasion, and other metabolic gene pathways were found using VFDB v.6.0 (10).

### Data availability.

This whole-genome shotgun project was deposited at DDBJ/ENA/GenBank under the accession number JAOBTH000000000. The version described here is JAOBTH010000000. The sequences are publicly available under the BioProject accession number PRJNA877611, BioSample accession number SAMN30712836, and SRA accession number SRR22752080. The GenBank accession number for the 16S rRNA gene sequence is OP381101.
